# Independent introductions and admixtures have contributed to adaptation of European maize and its American counterparts

**DOI:** 10.1371/journal.pgen.1006666

**Published:** 2017-03-16

**Authors:** Jean-Tristan Brandenburg, Tristan Mary-Huard, Guillem Rigaill, Sarah J. Hearne, Hélène Corti, Johann Joets, Clémentine Vitte, Alain Charcosset, Stéphane D. Nicolas, Maud I. Tenaillon

**Affiliations:** 1 Génétique Quantitative et Evolution – Le Moulon, Institut National de la Recherche agronomique, Université Paris-Sud, Centre National de la Recherche Scientifique, AgroParisTech, Université Paris-Saclay, France; 2 UMR 518 AgroParisTech/INRA, France; 3 Institute of Plant Sciences Paris-Saclay, UMR 9213/UMR1403, CNRS, INRA, Université Paris-Sud, Université d’Evry, Université Paris-Diderot, Sorbonne Paris-Cité, France; 4 CIMMYT (International Maize and Wheat Improvement Centre), El Batan, Texcoco, Edo de Mexico, Mexico; University of Minnesota, UNITED STATES

## Abstract

Through the local selection of landraces, humans have guided the adaptation of crops to a vast range of climatic and ecological conditions. This is particularly true of maize, which was domesticated in a restricted area of Mexico but now displays one of the broadest cultivated ranges worldwide. Here, we sequenced 67 genomes with an average sequencing depth of 18x to document routes of introduction, admixture and selective history of European maize and its American counterparts. To avoid the confounding effects of recent breeding, we targeted germplasm (lines) directly derived from landraces. Among our lines, we discovered 22,294,769 SNPs and between 0.9% to 4.1% residual heterozygosity. Using a segmentation method, we identified 6,978 segments of unexpectedly high rate of heterozygosity. These segments point to genes potentially involved in inbreeding depression, and to a lesser extent to the presence of structural variants. Genetic structuring and inferences of historical splits revealed 5 genetic groups and two independent European introductions, with modest bottleneck signatures. Our results further revealed admixtures between distinct sources that have contributed to the establishment of 3 groups at intermediate latitudes in North America and Europe. We combined differentiation- and diversity-based statistics to identify both genes and gene networks displaying strong signals of selection. These include genes/gene networks involved in flowering time, drought and cold tolerance, plant defense and starch properties. Overall, our results provide novel insights into the evolutionary history of European maize and highlight a major role of admixture in environmental adaptation, paralleling recent findings in humans.

## Author summary

The spread of a species outside its native range depends on its ability to face new environmental challenges. Despite a loss of diversity associated with recurrent introductions, domesticated species offer excellent examples of rapid expansion and adaptation to new climatic and ecological conditions. This is exemplified in maize, which was first domesticated in a restricted area of Central Mexico but now displays one of the broadest cultivated ranges of all crops. Here, we focused on the largely overlooked history of European maize, which was introduced from American sources. We sequenced 67 genomes from both continents. The data suggest two independent European introductions and also the admixed origin of three groups: maize from the US Corn Belt, European Flints and Italian material. We found modest genetic footprints of bottlenecks from European introduction. We detected signs of past selection at genes and gene pathways involved in adaptation to abiotic (drought/cold) and biotic (pathogens/herbivores) challenges. Our results provide novel insights into the evolutionary history of European maize and highlight a major role of admixture in environmental adaptation.

## Introduction

Expansion of species to conditions that differ from their native range depends on their abilities to adapt to new environments. Depletion of genetic diversity of introduced—founder—populations may however alter this ability. Despite loss of diversity associated with population bottlenecks during their domestication [1], domesticated species offer repeated examples of shifts in geographic range. There is in fact little overlap between the centers of origin of major crops and their area of highest production in the modern world [2]. For example, niche modeling of primitive maize landraces and cotton cultivars has demonstrated that their range rapidly exceeds that of their wild relatives [3,4]. Such rapid expansion is facilitated along east-west axes sharing more similar day-length characteristics and climates than along north-south axes [2]. Accordingly, data suggest that the evolution of vernalization or photoperiod-neutral requirements has delayed expansion of wheat and barley to Northern latitudes by hundreds of years [5].

In plants, while numerous studies have documented the early demographic and selective history of domesticated plants [6,7], a handful has focused on the routes of migration of crops outside their native range. It is clear, however, that migration routes and subsequent admixtures are important drivers of adaptation. For instance, adaptation of Tibetan highlanders to hypoxic conditions was facilitated through introgressions from individuals of Denisovan ancestry [8]. Similarly, introgressions from Neanderthal have contributed to functional adaptive variation at innate immunity genes in modern European populations [9]. In this study, we propose to address these issues in maize, a prime example of crop adaptive success displaying one of the broadest cultivated range.

Maize was domesticated around 9,000 BP in the tropical lowlands of Mexico [10,11]. Domestication and breeding bottlenecks in the Americas have reduced genetic diversity by 20% and <5% respectively [12]. Spread of maize landraces throughout the American continent has been addressed by earlier studies showing two major expansions northwards and southwards from Mexico [13]. In contrast with the well-established history of American maize, the history of European maize has been largely overlooked (reviewed in [14]). Two molecular studies, based on a restricted set of markers, have converged on the following conclusions. First, introductions to Europe occurred soon after the discovery of the Americas by Columbus. Second, landraces from northern Europe are related to northern American landraces, while landraces from southern Europe are related to Tropical lowland maize, which are of Caribbean origin [15]. Third, paralleling the emergence of the Corn Belt Dents at mid-latitudes in the US, the European Flints may derive from admixture between European northern and southern material [15,16].

Previous studies have genotyped maize from the Americas with MaizeSNP50k genotyping array [17], Genotype-by-Sequencing technology [18,19], or low-depth (5x) whole-genome sequencing [20] to detect genomic regions targeted by selection at the species level. More recently, Unterseer and colleagues [21] have undertaken an alternative approach by contrasting lines from two temperate heterotic groups (Dents and Flints) to screen for breeding signals using the MaizeSNP600k array that targets about half of maize annotated genes. Those studies have collectively identified hundreds of putative genomic targets of selection during ancient and more recent breeding history—in traditional landraces and elite maize lines. The phenotypic impact of a handful of genes has been validated through linkage-based cloning and association mapping, among which *ZmCCT* has been shown to contribute to day-length adaptation [22], and *Vgt1* [23] and *ZCN8* [24] are associated with flowering time. Because of the rapid decline of linkage disequilibrium in maize [1], a high density of markers is necessary to address a more complete inventory of genomic targets.

To document routes of introduction, patterns of admixture and the selective history of European corn and its American counterparts, we performed whole-genome sequencing of 67 maize lines from the two continents at mid-depth (18x). We purposely sampled inbred lines directly derived from landraces both to avoid the confounding effects of recent breeding selection and to account for geographical information. With >22 million SNPs identified, we (1) describe the genome-wide distribution of heterozygosity along chromosomes, (2) assess proposed sources of European maize, (3) measure the impact of introduction bottlenecks, (4) explore admixture events, (5) track signatures of selection at the level of genes and gene networks, using latitudinal and longitudinal contrasts.

## Results

With the purpose of tracing the origins of European maize and investigating its demographic and selective history, we combined genetic, historic and geographic information to select a sample of 67 maize lines. This sample is representative of European maize diversity and encompasses all possible American introduction sources. We specifically targeted lines directly derived from traditional populations (landraces), for which we have established the geographic origin from historical records. Such lines are less heterozygous than landraces and therefore more amenable for high-throughput sequencing (HTS) genotype calls. They are also less affected by recent and intense selection than elite lines, and therefore particularly relevant for conducting population genomic analyzes with a historical perspective.

**Fig 1:**
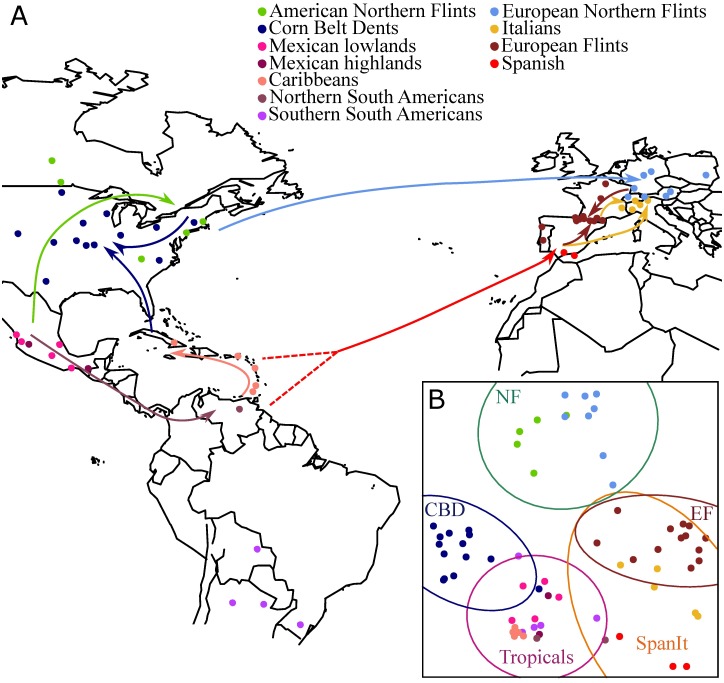
Sample locations, genetic structuring and inferred routes of maize migration. (A) Geographic location of 66 landraces from which lines originated with colors of dots designating genetic groups defined *a priori* (S1 Table)—a Spanish line for which the geographical coordinates are unknown is not represented. Arrows indicate inferred routes of maize migration with admixed groups displaying two arrows. Colors of the arrows correspond to the recipient group. (B) Principal Component Analysis computed on 500k non-genic SNPs. Samples were assigned to 5 genetic groups defined by FastStructure (S1 Table). Corresponding ellipses indicate the 95% CI of the Mahalanobis distance.

Altogether our sample included 48 first-cycle maize inbred lines, 9 lines obtained by single seed descent and 10 doubled haploids. The lines covered 11 major groups described in previous studies [13,25] and thus defined here *a priori* (Fig 1A, S1 Table). These eleven groups are the American (n = 5) and European (n = 8) Northern Flints (ANFs and ENFs), which we collectively call the Northern Flints, European Flints (EFs, n = 13), Spanish (n = 3), Italians (n = 5), Mexican lowlands (n = 5) and highlands (n = 2), Caribbeans (n = 5), Southern (n = 5) and Northern South Americans (n = 2), and Corn Belt Dents (CBDs, n = 14).

### Polymorphism discovery and accuracy of genotype calling

We generated 2x100 bases paired-end reads from High Throughput Sequencing (HTS) of 67 lines totaling 2,654 Gb of sequences. We aligned them to the B73 v2 reference genome [26]. The percentage of paired-end reads mapped with correct insert sizes ranged between 68.2% and 80.1%, depending on the line (S1 Table). Considering only bases covered by at least 4 reads (4x depth), the per-line reference genome coverage varied between 70.3% and 78.6% (S1 Fig).

The maize genome has undergone several rounds of duplications and contains large proportions (85%) of transposable elements [26]. This genomic redundancy makes polymorphism calling a challenging task. In particular, misalignment of paralogous regions often causes heterozygotes miscalling. We designed customized filters to increase the accuracy of SNP calling. A small proportion of positions called by Varscan (~16%) passed these filters. Among positions that we discarded: ~30% contained > 50% of missing data; ~30% became monomorphic after filtering for read-depth variation, multiple mapping, and genotype uncertainties (LRT not significant); and another ~40% were suspected to belong to duplicated regions based on error count, heterozygotes count, elevated read-depth variation and/or proportion of multiple mapped reads.

We obtained a dataset of 22,294,769 Single Nucleotide Polymorphisms (SNPs) after filtering, of which 86.0% were located outside genes, 5.4% in exonic regions and 8.6% in intronic regions. In total 34,350 out of the 39,423 genes of the maize genome were covered by at least one SNP. The percentage of missing data per line ranged between 18.2% and 32.1% (S2 Fig). 2,189,230 (9.8%) SNPs encompassed no missing data. Given genome divergence between species, we were able to map only 6.19% of HTS data from *Tripsacum dactyloides* [20] to the B73 v2 reference genome. In total, we managed to orientate 1,255,761 of our SNPs with *Tripsacum dactyloides*, half of which (48.5%) were located in genic regions.

Proportion of heterozygous sites (SNPs x lines) was initially comprised between 26.5% and 37.5%, but decreased after filtering to a range of 0.9% to 4.1% (S1 Table). To test the efficiency of our filters to remove false heterozygotes, which are often confounded with homozygous allelic variants at duplicated sites, we compared the average heterozygous sites proportion across lines between a set of genes encompassing paralogs (2,788 genes) and a set of single genes with no paralogous copy (3,949 genes) [27]. We found no significant difference between the two sets with a two-sided Kolmogorov-Smirnov test (P-value = 0.873), thus indicating that gene paralogy is well accounted for heterozygous calls.

We further estimated miscalling of both homozygotes and heterozygotes by comparing our HTS genotype calling to that obtained with the MaizeSNP50k genotyping array (S1 Table, ~38,000 positions analyzed). We found a rate of discrepancy between the two datasets that ranged from 0.016% to 0.21% per line—with an average value of 0.038% across lines. For a subset of 20 lines (S1 Table), our MaizeSNP50k and HTS genotypes were obtained from the same DNA extractions. Considering this subset, the error rate of genotype calling in our HTS data for homozygotes dropped to 0.036%. In contrast, for heterozygotes (181 positions on average per individual), the percentage of false negatives—proportion of homozygotes in our HTS data among heterozygotes in the MaizeSNP50k data—and false positives—proportion of false heterozygotes among heterozygotes in our HTS data—was 18.3% and 34% respectively.

Finally, we assessed the power of our HTS approach over MaizeSNP50k SNP to analyze maize genetic diversity by comparing folded Site Frequency Spectra (S3 Fig). The two SFS differed markedly, pointing to a marked deficit of rare variants in the array data. Such a deficit is expected, because a restricted panel of lines was used for SNP discovery for the MaizeSNP50k SNP design.

In sum, our HTS approach combined multiple advantages: high SNP density; a low error rate for homozygous calls; and a realistic description of allele frequencies, including low frequency variants that are likely to have arisen recently and are therefore informative for assessing both recent ancestry and selection.

### Patterns of genetic diversity reveals a complex demographic history

**Fig 2:**
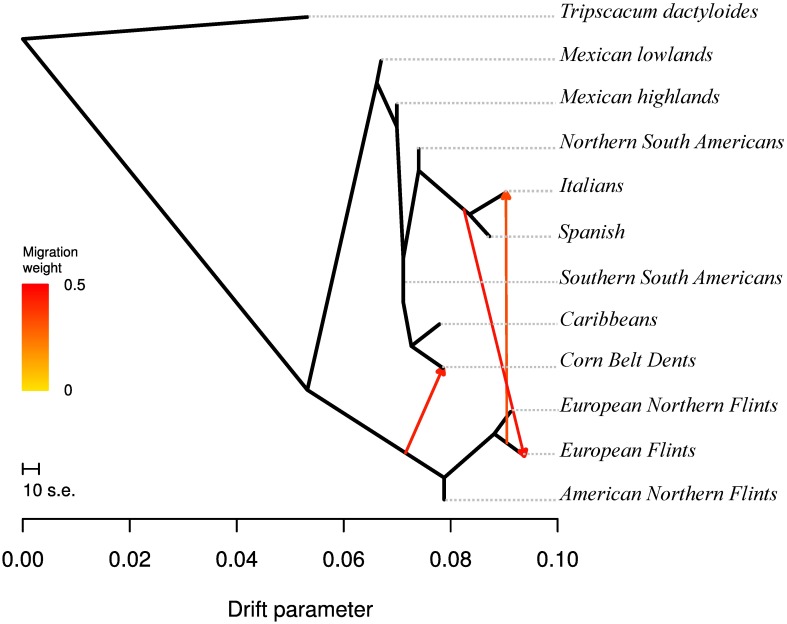
Historical splits and admixtures between populations as inferred by TreeMix using a model with 3 admixture events. The TreeMix model explains 99.75% of the variation. Admixtures are colored according to their weight. The model indicates an admixed origin of Corn Belts Dents (arrow weight = 0.44), the European Flints (arrow weight = 0.47), and the Italian material (arrow weight = 0.35).

In order to get insights into the history of divergence and admixtures in our sample, we applied Treemix to the 11 groups that were defined *a priori* (S1 Table), using *Tripsacum dactyloides* as an outgroup. The corresponding unfolded SFS is shown in S4 Fig. As illustrated in Fig 2, our results showed: (1) a major split isolating Northern Flints and European Flints from the rest; the marked distinction of NFs from ancestral Mexican lines was confirmed by elevated Fst values between NFs and all other groups (S2 Table); (2) proximity of NFs and EFs, the latter being admixed by ancestors of the Southern European Material; (3) an admixed origin of the CBDs between a Northern Flint ancestor and tropical material sharing a recent ancestor with the Caribbeans; (4) an admixed origin of the Italian material between Southern European material and EFs (Fig 2).

We further tested admixed origins of EFs, CBDs, and Italians by computing three-population tests (*f3*) with groups that are the most closely related to putative sources—ENFs and Spanish lines for EFs, ANFs and Caribbeans for CBDs, EFs and Spanish for Italians). We found significant negative values for EFs and CBDs, confirming their admixed origins (EFs admixture, *f3* = -0.009, Z = -21.09, P-value<10^−10^; CBDs, *f3* = -0.005, Z = -13.87, P-value< 10^−10^). Results for Italians were more equivocal (*f3* = 0.0005, Z = 3.64, P-value<2.7 10^−4^).

FastStructure analysis revealed a model maximizing the marginal likelihood at *K* = 4, with the four groups defined by Northern Flints (NFs), European Flints (EFs)+ Italians, Corn Belt Dents (CBDs), and Spanish + Tropicals (S5A Fig). However, at *K* = 5, both Spanish and Italian (SpanIt) lines formed an independent cluster (S5B Fig). Inspection of PCA analysis with ellipses at *K* = 5 revealed that the first two axes mirror geographical origins of lines, and a clear distinction of the NFs (from America and Europe) from the remainder of the sample (Fig 1B). The CBDs as well as SpanIt clearly displayed shared ancestry with Tropicals, while EFs displayed genetic proximity with SpanIt. Note that Tropicals regrouped material from Mexico (lowlands and highlands), South America (northern and southern) and Caribbeans. Grouping of Tropicals was further supported by extremely low Fst values (<0.05), with an average Fst of 0.02 within Tropicals, i.e. average value obtained from all pairwise comparisons involving material from Mexico, South America, and Caribbeans as listed above. In comparison, we obtained an average Fst of 0.15 when comparing Tropicals to all other groups (S2 Table). We estimated genome-wide nucleotide diversity (*π*/bp) of the non-genic compartment for the 5 genetic groups: Tropicals displayed the highest level of genetic diversity of all 5 groups (0.00824, n = 19), followed by EFs (0.00739, n = 13), CBDs (0.00736, n = 14), SpanIt (0.00694, n = 8), and NFs (0.00648, n = 13). Distributions of nucleotide diversity for all genetic groups are shown in S6 Fig.

Finally, we performed a more detailed inspection of admixture of CBDs and EFs using FastStructure. While American Northern Flints contributed less to the CBDs than the Tropicals (S7A Fig), the contribution of NFs and Southern European material to the EFs was balanced (S7B Fig).

### Footprints of European introductions

**Fig 3:**
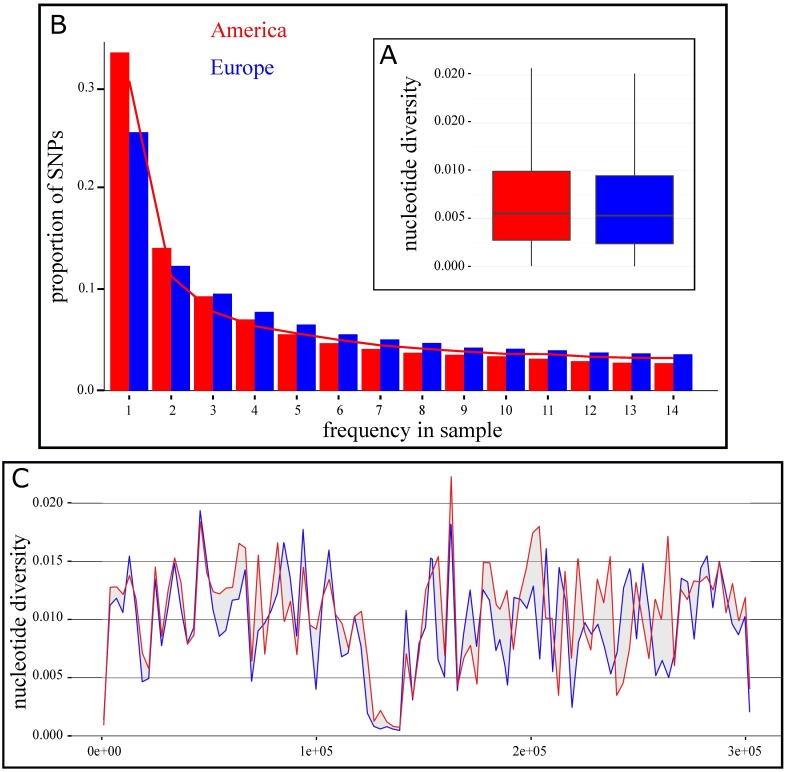
Genome-wide patterns of nucleotide diversity of American and European samples. A: Box-plots of non-genic per-bp nucleotide diversity (*π*) estimated on 4,799 non-overlapping segments of 10kb along the genome. B: Folded Site Frequency Spectra of the European sample and the American sample projected down to 29 samples. SFS were built on a common set of 2,941,528 non-genic SNPs. SFS expectation for American landraces from a model incorporating a domestication bottleneck, population expansion and gene flow (parameters from [28]) is shown by a red line. C: Variation of *π*/bp along chromosome 1 computed from 50kb sliding windows.

One of our main goals was to test whether introductions of European corn were associated with a bottleneck. We first examined the Site Frequency Spectrum (SFS) of our American sample and compared it to the expected SFS under the most recent demographic model proposed for American maize (Fig 3B). We found a close match between the observed and predicted SFS. Second, we searched for footprints of a European bottleneck, which may include a loss of diversity, a skew of SFS towards loss of rare variants, which should translate into an increase of Tajima’s *D*.

We found subtle footprints of this introduction bottleneck: (1) a decrease in nucleotide diversity indexes, *π* and *θ*, at non-genic SNPs (*π* = 0.00842 and 0.00783 and *θ* = 0.00926 and 0.00736 for American and European samples respectively), which represented a significant loss of diversity in Europe (Fig 3A, P-value = 1.39 10^−11^); (2) an increase in *D* at non-genic SNPs (*D* = -0.480 and 0.111 for American and European material, respectively); (3) a deficit of rare variants in the European material compare with the American material (Fig 3B). Genome-wide diversity along chromosome 1 as illustrated in Fig 3C (and S8 Fig for all other chromosomes) reveal congruent patterns between the American and European material.

### Differential selection and association with latitudinal and longitudinal variation

Structure analyses identified 5 genetic groups (S5 Fig) distributed along two longitudinal axes (corresponding to the two continents, Fig 1B). Both latitude (within continent) and longitude (between continents) are primary determinants of climate variation. We employed two complementary approaches to search for genic targets of selection associated with climate adaptation. First, we combined signals of differential selection between groups and positive selection within groups to identify Differentially-Selected candidates (DS_candidates). We performed 3 latitudinal comparisons within the American continent (NFs/CBDs, CBDs/Tropicals, NF/Tropicals) and 3 latitudinal comparisons within the European continent (NFs/EFs, EFs/SpanIt, NFs/SpanIt). We next performed 3 longitudinal comparisons between continents (ANFs/ENFs, CBDs/EFs, Tropicals/SpanIt).

In this differential selection approach, we employed the non-genic data as a control for forces other than selection, which includes demography and regional genomic variation in mutation and recombination rates. We screened the genome for selective footprints using a combination of 3 statistics: Fst and XP-CLR that detect extreme differentiation of allele frequencies between groups (the latter accounting for local linkage disequilibrium), and *D* that detects selection within a group. Genes passing the 5% threshold for all 3 statistics were considered as candidate genes.

Among 9 pairwise comparisons (Table 1) we detected 968 DS_candidate genes (S3 Table), and 252 of them were involved in multiple comparisons. That a given gene was involved in multiple comparisons was mainly caused by a given group being used in multiple comparisons rather than a signal of convergent selection between comparisons using distinct groups. The number of DS_ candidates varied between 46 and 219 (Table 1) among comparisons, with the CBDs and Tropicals comparisons offering the smallest number of candidates. On average, latitudinal comparisons yielded 155 candidates and longitudinal comparisons yielded 129. Interestingly, while ANFs and ENFs are genetically closely related and grow at similar latitudes, their contrast exhibited high numbers of candidate genes for selection (147). Patterns of selection within groups (Table 1) also differed, with NFs displaying repeated signals of positive selection (respectively 112, 124, 132, 125 depending on the comparison), while CBDs exhibited a more modest number of candidates when compared with other American groups (56 with the NFs and 21 with the Tropicals).

**Table 1:** Number of genes exhibiting patterns consistent with Differential Selection (DS_candidates) in each pairwise comparison.

G1^a^	G2^a^	# cand, G1/G2^b^
Latitudinal comparisons		
NF	CBD	163 (112/56)
CBD	Tropical	46 (21/25)
NF	Tropical	175 (124/61)
NF	EF	201 (132/69)
EF	SpanIt	129 (50/81)
NF	SpanIt	219 (125/108)
Longitudinal comparisons		
ANF	ENF	147 (85/65)
CBD	EF	122 (81/51)
Tropical	SpanIt	119 (13/106)

^a^ Name of the genetic groups considered (number of individuals): NF(13), CBD(14), Tropical(19), EF(13), SpanIt(8), ANF(5), ENF(8).

^b^ Number of candidate genes detected as differentially selected in the comparison (within Group 1/within Group 2 as determined by *D*). The number of candidates may outnumber the sum of candidates in G1+G2 when differential selection is associated with positive selection within the two groups.

We assessed for each pairwise comparison the proportion of non-genic windows (used as controls) that passed the 5% threshold of all 3 statistics. Depending on the comparison, the proportion varied between 0.072% and 0.210%, with an average of 0.134%. Considering these proportions are representative of the expectation under a null hypothesis, we multiplied them by the total number of genes tested in each comparison and obtained between 13 and 74 DS_candidates. These numbers provide estimates of the expected number of false positives. However, because non-genic windows probably contain selected features, they are likely overestimated. Even so, Table 1 revealed an enrichment of DS_candidates for all pairwise comparisons. Note that if we considered each statistic independently, the proportion of genes passing the 5% threshold over all pairwise comparisons was below 5% in 80% of the cases. In other words, the actual test level was below the nominal level in most cases, indicating that our approach was conservative.

In the second approach, we considered latitude and longitude as quantitative phenotypes in a Genome-Wide Association (GWA) framework to test for the effect of individual genic SNPs on latitude/longitude while accounting for group membership *i*.*e*. structure (S1 Table) and relatedness among lines, *i*.*e*. kinship. We used here the entire set of lines. This approach differs fundamentally from the differential selection approach in several aspects: it considers a phenotype (latitude), it accounts for inter-group effects and reveals intra-group effects, *i*.*e*. only genes with consistent effects across groups are detected. We found 401 and 424 GWA_candidates associated with latitude and longitude respectively, with 42 genes in common between DS_candidates and GWA_candidates (S4 Table).

### From candidates to gene ontology to selection along gene networks

**Fig 4:**
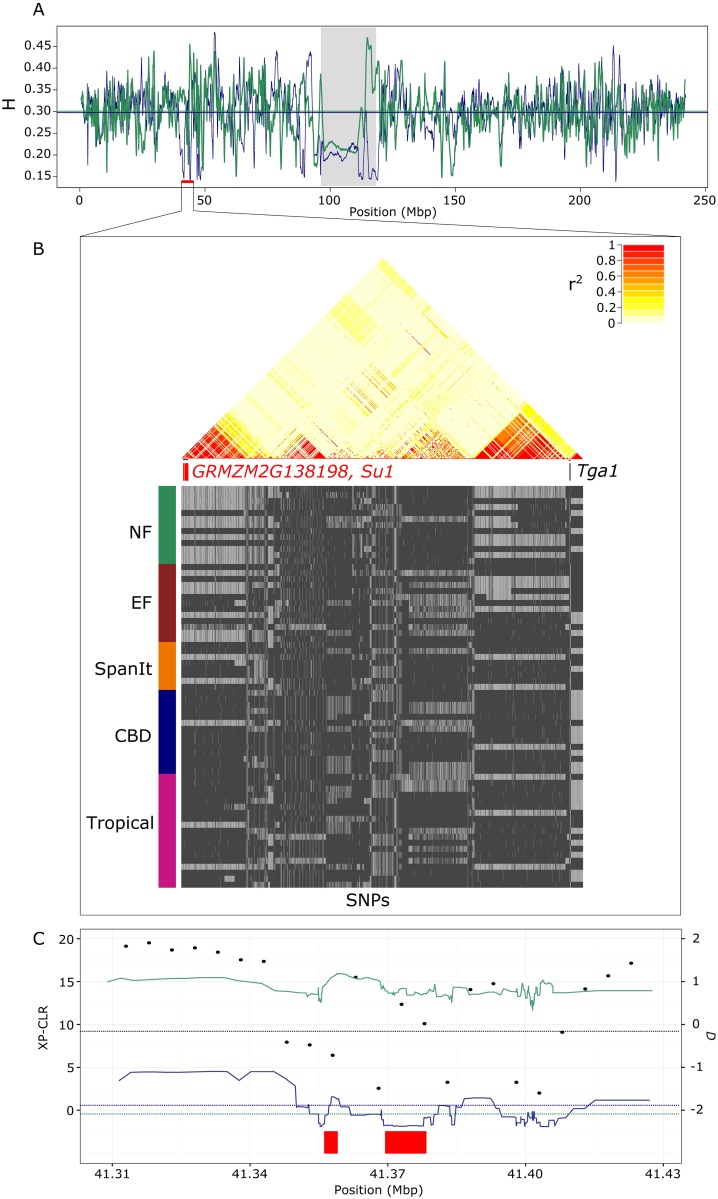
Patterns of differential selection at *SU1* gene and the nearby *TGA1* domestication gene. (A) Nei’s genetic diversity (H) averaged on overlapping sliding windows of 1,000 SNPs (1 SNP step) along chromosome 4 with positions indicated in kb (CBDs in dark blue, NFs in green). (B) Status of biallelic SNPs per line within each genetic group as inferred from Structure is shown (with most frequent allele in dark grey) as well as pairwise LD computed from r^2^ from positions 41,000,000 to 45,001,000 bp. Gene positions *SU1*, *GRMZM2G138198* are indicated with boxes in red (candidate genes) and *TGA1* in black. (C) Zoom within the region with XP-CLR (dots) and *D* values (lines with the same color code as in A) presented along with the genome-wide 5% quantile (dark blue and green dotted line for *D* within CBDs and NFs respectively, and black dotted line for XP-CLR between CBDs and NFs). *GRMZM2G138198* and *SU1* positions are indicated by red boxes.

An example of a DS_candidate is illustrated in Fig 4. Patterns at *SU1* revealed reduced diversity in the CBDs as compared with the NFs (Fig 4A), and XP-CLR values deviating from the genome-wide estimates (Fig 4C, S3 Table). Consistently, one main haplotype segregated around *SU1* within CBDs and to a lesser extent within Tropicals, with an overall elevated level of LD within this region (Fig 4B). Selection within CBDs was further confirmed by significantly negative *D* values (Fig 4C, S3 Table). Note also selective footprints in the region surrounding the nearby domestication gene *TGA1*, as illustrated by elevated LD with the segregation of two major haplotypes (Fig 4B).

Association between latitude and allele frequency was found at *GRMZM2G095955* (GWA_candidate, S4 Table), a gene located in the vicinity of maize floral activator, *ZCN8* [29]. Patterns in the *ZCN8* region revealed a haplotype common to all temperate material and the segregation of this “temperate” haplotype with a “tropical” haplotype within Tropicals and to a lesser extent within CBDs (S9 Fig).

Along with previously characterized genes, we also revealed new candidates such as *ZCN5*, which is also known as *PEBP5*. This gene harbored strong evidence of selection both within the Tropicals (S10C Fig) and within the European Flints when compared with the NFs with corresponding significant negative *D* values (S3 Table). A specific haplotype for these groups differed markedly from the most common NF haplotype (S10B Fig). Interestingly, both haplotypes segregated at intermediate frequency within the CBDs (S10B Fig), as denoted by a significant positive *D* (S3 Table, S10C Fig).

Considering candidate genes detected among all pairwise comparisons in both DS_ and GWA_ approaches, we found significant enrichment in 19 MapMan ontologies (S5 Table). We further grouped MapMan ontologies into 23 main categories, and found enrichment in 2 out of the 23 main categories: carbohydrate metabolism, proteins (S5 Table).

We also tested for a global enrichment of differential selection signals (using Fst) at the gene network level rather than at the individual gene level, following a method proposed by [30]. In this purpose, we used 294 maize gene networks described in the MaizeCyc database. We found 62 significant pairwise comparisons at α <1%, corresponding to 44 gene networks. Because some gene networks had more than 50% of genes in common, we grouped genes into 25 network clusters (S6 Table). Among network clusters with a potential role in adaptation, we found signs of selection on the abscissic acid (ABA) biosynthesis network (Network Cluster #2, S6 Table), the putrescine pathway (Network Cluster #9, S6 Table), the β-caryophyllene biosynthesis pathway (Network Cluster #11, S6 Table), the *cis*- and *trans*- zeatin biosynthesis pathways (Network Cluster #18, S6 Table).

### Genome-wide distribution of residual heterozygosity

We developed a segmentation method to detect regions with patterns of heterozygosity that deviate from genome-wide expectations, *i*.*e*. heterozygous segments. We applied our method to the subset of 57 first-cycle inbred and SSD lines. Deviations were detected either because heterozygosity extended over an unexpectedly high number of adjacent sites (in a single or multiple lines) or because heterozygosity was unexpectedly conserved across lines (at a single or multiple sites). We retained 17,959 segments genome-wide, each encompassing at least 5 SNPs. Among these, 6,978 exhibited significantly elevated heterozygosity relative to that of the entire genome (0.41%) as determined by an exact Bernoulli test procedure followed by an FDR control at a nominal level of 5% (S7 Table). Heterozygous segments encompassed/overlapped with 4,354 annotated genes (S7 Table). They covered a total of 166,892,128 bases. No particular distribution pattern was observed at the chromosome level (S11A Fig).

An example of one of the most significant regions (chr. 6,162,120,176–162,124,810 with a P-value<10^−6^, S7 Table) is provided in S11B Fig. Within this region, we detected one heterozygous segment encompassing 20 SNPs displaying conserved heterozygosity in over 33% of lines (on average). This heterozygous segment overlapped with the gene *GRMZM2G174990* (S7 Table). In addition, we detected a heterozygous segment (162,126,864–162,133,490, P-value = 6.10^−6^) spanning 10 SNPs and 12 heterozygous genotypes. A single line was heterozygous for 5 of the 12 SNPs, and 7 other lines were heterozygous for 1 of the 12 SNPs. This heterozygous segment overlapped with two genes, *GRMZM2G174949* and *GRMZM2G174971* (S7 Table). Note also a cluster of 19 SNPs located in a region spanning 4,170,746 bp on chromosome 6 (9,891,618–14,062,364 with a P-value<10^−6^, S7 Table and S11A Fig). On average 14.9% of lines were heterozygous in this region. It encompassed 37 genes (S7 Table).

To further investigate the origin of these heterozygous segments, we used Doubled Haploids (DHs). By definition, detection of heterozygosity in DHs may only be explained by alignments artifacts. DHs hence provide an opportunity to test the relative contribution of reads misalignment *versus* allelic variation to heterozygous segments. We tested significance of heterozygous segments originally detected from our subset of 57 lines in the DHs subset (6,978 heterozygous segments of which 5,069 contained at least 5 SNPs in DHs, S7 Table). The distribution of P-values obtained from DHs revealed a mixture of two distributions (S12 Fig): one resembling a uniform distribution as expected under the null hypothesis, which corresponded to segments not significant in DHs (class 1); the other with an excess of small values, containing significant segments in DHs (class 2). Class 2 encompassed ~21% of heterozygous segments (1,088 out of 5,069, S7 Table). These segments corresponded most probably to alignment artefacts caused by underlying structural variants. Interestingly, the distribution of the number of contributors to heterozygous segments differed markedly between the two classes. While heterozygous segments (6,978) discovered in our subset of 57 lines was dominated by the contribution of one or two contributors (S13A Fig), the subset of segments significant in DHs (1,088) displayed instead a majority of conserved segments across multiple lines (S13B Fig).

We used SIFT [31] to predict deleterious amino acid substitutions throughout the genome. SIFT is based on conservation of amino acids within protein families to classify substitutions as tolerated or not tolerated, *i*.*e*. deleterious. Interestingly, heterozygous segments encompassed significantly more deleterious mutations than the rest of the genome. Genic SNPs outside those segments contained 7.07% of predicted deleterious variants *vs* 7.57% within segments (χ^2^ = 23.78, d.f. = 1, P-value = 1.07 10^−6^).

## Discussion

During its spread through the Americas and subsequent introduction to Europe, maize has adapted to a vast range of latitudes. Here we have investigated how distinct migration routes and admixture histories have contributed to shape maize genetic diversity and adaptation on the two continents. For this purpose, we selected an original sample of 67 lines that were directly derived from landraces. These lines have not been affected by modern breeding, and are therefore suitable to address questions about evolutionary history prior to extensive modern breeding. We have generated whole genome sequencing to produce a dataset of SNPs with reduced ascertainment bias and allele frequencies that include low frequency variants (S4 Fig).

Maize is characterized by high nucleotide diversity (10–20 times greater than humans [32]), a vast amount of structural variation [33], and extended paralogy [34]. Our methodology has accounted for those specificities and allowed accurate detection of homozygous SNPs despite the complexity of the maize genome. Our estimated miscall rate of 0.036% is about 8 times lower than that estimated from 5x whole genome sequencing data (0.31%, [20]). We genotyped over 22 million SNPs that covered 87% of annotated genes, providing the largest whole genome sequencing effort to date of the maize European germplasm.

### Characterizing regions with unexpected patterns of heterozygosity

Heterozygotes are much more challenging to call than homozygotes particularly with coverage <20x [35]. Consistently, we found a much higher rate of false positives (34%) for heterozygotes. This rate still compares advantageously to previous estimates from 103 lines, which had a false positive rate of nearly 75% [20]. A small proportion of heterozygotes miscalling may be explained by genotyping errors in the data generated by the MaizeSNP50 array. We indeed found examples of array-called heterozygous SNPs in our sample of 3 doubled-haploids (0.16% of genotypes on average).

Although heterozygotes calling in our HTS dataset was error prone, we have postulated that extended segments of heterozygosity (along a region or among lines) provide evidence for either mapping artifacts caused by underlying structural variants (duplications) or regions that are resistant to homozygosity. Selection against allele combinations that are highly deleterious when homozygous may indeed help overcoming inbreeding depression. Previous studies have proposed that elevated rate of heterozygosity in low-recombining pericentromeric regions of recombinant inbred lines is a consequence of inefficient purging of deleterious alleles [36,37]. In contrast, we have observed no specific patterns of heterozygosity along the chromosomes (S8 Fig).

Because deleterious alleles segregate at low frequencies in the maize genome, they are difficult to use in association mapping frameworks that combine genotypes and phenotypic measures of inbreeding depression [38]. The power of our segmentation approach is to rely on repeated evidence, either among lines or regions, to discover regions of unexpectedly high heterozygosity. Using a subset of 57 first-cycle inbreds and SSD lines (out of 67), we identified 6,978 segments of unexpectedly high heterozygosity rate. Together, these heterozygous segments represent 8.8% of all base pairs analyzed. A substantial proportion of heterozygous segments (~21%) also displayed an elevated rate of heterozygosity in our subset of 10 doubled haploids, consistent with alignment artefacts caused by underlying structural variants. On chromosome 6, for example, we found a segment of extended heterozygosity bearing 37 genes (S11A Fig), 6 of which are reported as part of the highest read depth variants in maize [20], signaling a structural variant recently confirmed by PCR assays in a collection of lines [39]. But the majority of our heterozygous segments were not detected in doubled haploids and may therefore point to inbreeding depression candidates. While the difference is small, we actually found significantly more deleterious variants in heterozygous segments (7.57%) than in the rest of the genome (7.07%). Altogether, our results suggest that selection against inbreeding depression has played a role in the maintenance of residual heterozygosity in our sample of maize lines.

### American sources and footprints of European introductions

In the Americas, two historical major expansions northwards and southwards from Mexico have been documented [13] (Fig 1). The northwards expansion through southwestern US to the northern US and Canada gave rise to the Northern Flints [40,41]. Introduced around 500 BC, Northern Flints gradually became the main crop in eastern North America [42]. Southern Dents appeared 2000 yrs later and likely derived from southeastern US introductions with influence of Caribbean flints [43,44]. Corn Belt Dents (CBDs), that are adapted to the Midwestern US climate, emerged about 200 years ago from crosses between southern Dents and Northern Flints [43]. Our data confirmed their admixed origin (S7A Fig) but also pointed to a greater contribution of Southern material. Interestingly CBDs in the Treemix analysis (Fig 2) appeared to share a close ancestor with the Caribbean rather than with Mexican material, perhaps mirroring the Caribbean contribution to the Southern Dents [43].

We have combined evidence from inferred genetic proximities and admixture events revealed by Treemix as well as historical records to propose a model of maize migration in Europe (Fig 1). Our data are consistent with two major introductions in Europe. First genetic proximity between European and American Northern Flints (ANFs and ENFs) indicate that the latter derived from the former (Fig 2). Second, the Spanish materials appear to share a common ancestor with the South American material (Fig 2). The latter observation questions the Caribbean origin of Spanish maize. Because there is clear evidence that Columbus imported maize from Cuba to Spain by 1493 [45], it is possible that our limited sample did not capture the source of the Spanish material but rather the South American origin of the Caribbean material subsequently introduced in Southern Europe.

Regarding a putative third independent introduction of the Italian material from Argentina [16], our results were more consistent with the emergence of Italian lines from an admixture between Spanish and European Flints (Fig 2). Letters actually attest to the donation of maize from Spain to the Vatican in Rome after the second voyage of Columbus as early as 1494 [46]. Finally, we have substantiated—with a much larger dataset than [15,16]–the admixed origin of the European Flints (EFs) from two contributors, the ENFs and the Tropicals.

Altogether our results show that while genetic diversity is significantly lower in Europe than in America (Fig 3A & 3C), European introductions left only modest footprints on the distribution of allele frequencies (Fig 3B). We found evidence for two independent introductions to Europe and propose that adaptation to mid-latitudes both in the US (CBDs) and Europe (EFs and Italians) has depended on admixture.

### Screening for genic targets of selection along latitude and longitude

Our sample encompassed 5 genetic groups (Fig 1B and S5B Fig) distributed over a broad range of latitudes along two longitudinal axes (the two continents). Both latitude and longitude are primary determinants of climate variation. However, because locations at the same latitude share identical day-length and seasonalities and often similar climates [2], we expect latitudinal contrasts to reveal more adaptations than longitudinal contrasts. Consistently, among Differentially Selected candidates (DS_candidates) we have observed on average more candidates in latitudinal contrasts (155 genes) than in longitudinal contrasts (129 genes). However, the opposite trend was observed in the GWA analysis (401 versus 424), perhaps highlighting the role of other adaptive forces such as habitats and biotic components. The number of DS_candidates detected for the Northern Flints (>112 in all comparisons, Table 1) outnumbered those detected for the Tropicals (<61 in all comparisons), signaling more pervasive adaptation outside latitudes where maize originated.

In total, we retrieved 968 DS_candidates. In order to control the rate of false positives, we based our DS_candidates identification on genome-wide extreme values for three metrics (CLR test, *Fst*, *D*) that detect different hallmarks and time scales of selection [47]. While our approach was conservative, the comparison with the recent work of Unterseer and colleagues [21] that employed a similar methodology to detect DS_candidates revealed a greater number of common DS_candidate genes between the two studies (93, S3 Table) than expected by chance (P-value<2.10^−3^). Unterseer and colleagues [21] have contrasted Flint elite lines—grouping EFs and NFs from Europe—to Dent elite lines developed in Europe and the US, and accordingly most common DS_candidate genes (59 among 93) originated from our Flints and Dents comparisons (EFs/CBDs and NFs/CBDs). While significant, the overlap between the two studies was overall limited: first because the two studies differ in the time scale analyzed, recent breeding (elite lines were included in [21]) versus more ancient adaptive events represented by the first-cycle inbred lines in our study; second because we included tropical materials, thereby expanding our ability to find loci involved in the first steps of adaptation towards northern latitudes. More generally, although false positives may contribute to differences between studies, our results are consistent with the recruitment of distinct genetic mechanisms in different genetic groups.

Because polygenic adaptation may involve a collection of mutations with small effects, which collectively have a large effect on a given pathway, we adapted a gene-set enrichment test [30] to uncover genome-wide signals of differential selection along 294 gene networks. We found evidence for differential selection at 25 candidate gene network clusters (S5 Table). Overall, common signals of selection detected at individual genes and along gene networks were scarce. In fact, while our networks together encompassed 627 genes, only 16 genes that were detected as candidates by the gene-by-gene approach also belonged to selected networks (S3 Table). This result may illustrate potential complementarity of these two approaches, with the former detecting genes with potential strong allelic effect, while the latter aims at detecting polygenic adaptation emerging from selection of a multitude of alleles with small effects.

### Multifarious adaptation of maize

While a small fraction of our candidates has been functionally characterized in maize (S3 and S4 Tables), we can point to a number of interesting examples. For instance, we found a significant association between a polymorphism located in the vicinity of *ZCN8* and latitudinal variation (S9 Fig). *ZCN8* is the main floral activator of maize [29], and it is strongly associated with flowering time variation [48]. Here, patterns at *ZCN8* were consistent with segregation of two haplotypes in the Tropicals, and elimination of the late flowering haplotype from northern latitudes (late flowering haplotype displayed in light grey, S9 Fig). With a single exception, the late flowering haplotype was also counter-selected within CBDs and within EFs after admixture events in North America and Europe respectively. Interestingly, we also found signals of positive selection *ZCN5* (also known as *zen1* and *pebp5*), a gene from the same family as *ZCN8*. Here the “tropical” haplotype present in Tropicals strongly differed from the major “NF” haplotype (S10 Fig). In contrast to CBDs that exhibited the two haplotypes at intermediate frequency, the pattern in EFs was consistent with strong positive selection of the “tropical” haplotype in this group (S10 Fig). *ZCN5* is expressed in developing ears and tassels after floral transition in maize but its function remains undetermined [49]. While it is beyond the scope of our paper to perform functional validations, a recent study has reported an association between polymorphisms at *ZCN5* and flowering time variation [50].

Besides flowering, which is an obvious target of selection, we have also uncovered an important role for abiotic stress tolerance in maize adaptation. We have found significant enrichment for the tetrapyrrole synthesis category both in our gene-by-gene (S5 Table) and network approaches (S6 Table), as well as evidence of selection along the ABA synthesis gene network in ANFs/ENFs comparisons (S6 Table). One of our candidates, the *ZmASR2* gene (Abscisic acid-, Stress-, and Ripening-induced protein 2, *GRMZM5G854138*, S3 Table) actually displays increase in expression at the transcript and protein level under water deficit conditions [51]. Both ABA and tetrapyrroles entail drought tolerance via independent stimuli, cellular changes—loss of turgor—for the former, and ROS-mediated stress signaling for the latter [52]. Cold tolerance also contributed to maize spread as manifested by selection along the putrescine pathway (S6 Table)—and the largely redundant arginine degradation III arginine decarboxylase-agmatinase pathway—in multiple pairwise comparisons. The putrescine pathway is part of the polyamine metabolism activated in response to various abiotic stresses [53]. In *Arabidopsis thaliana*, putrescine controls ABA level in response to low temperature, thereby contributing to cold acclimation [54]. Along the same lines, we have revealed strong evidence of selection along *cis*- and *trans*-zeatin pathways in pairwise comparisons involving NFs *vs* either Tropicals or SpanIt (S6 Table). The zeatin biosynthesis pathways mediate responses to biotic and abiotic environmental interactions [55]. They are considered essential cytokinins and in conjunction with ABA are involved in various stress responses including cold, drought, and osmotic stresses [56].

Biotic factors are equally important contributors to maize adaptation and spread. We have found footprints of selection along the β-caryophyllene biosynthesis network in comparisons involving EFs versus Tropicals or CBDs (S6 Table). β-caryophyllene is a secondary metabolite that serves as foraging cues for natural enemies of herbivores [57]. The pathway includes the *TPS23* gene (*GRMZM2G127336*) responsible for the synthesis of a volatile sesquiterpene that attract natural enemies of herbivores upon release [58]. Interestingly, evidence from 24 North American lines suggest that the majority (22 out of 24) have lost the capacity of synthetizing this component [59], while the gene is actively transcribed in European material. Consistent with these observations, our results revealed selection within EFs at *GRMZM2G127336* (S3 Table). Likewise, selection at 6 genes from the *WRKY* family (S3 and S4 Tables) support the adaptive contribution of DNA binding transcription factors that regulate plant defense in response to various infections such as *Trichoderma* root colonization [59] and fungal pathogens [60]. For instance, expression of the putative *Arabidopsis thaliana* ortholog of *WRKY41* (S3 Table), *AtWRKY46*, is strongly induced by pathogen effectors, and therefore likely involved in the transcriptional reprogramming that initiate effector-trigger immunity [61].

In addition to phenology, abiotic and biotic stress responses, patterns at the *SU1* gene (*GRMZM2G138060*, S3 Table and Fig 4)—that encodes a starch debranching enzyme—uncovered differential selection on kernel phenotypes between NFs and CBDs [62]. In conjunction with other starch biosynthesic components [63], *SU1* contributes to the structure of amylopectin as well as the ratio of amylose over amylopectin, which affects gelatinization properties and texture [64]. *SU1* was targeted by selection during domestication [64]. Moreover, previous work has demonstrated gradual allelic selection at *SU1* from teosintes to early maize and to modern varieties [65]. The region bearing *SU1* and *TGA1*, one of the key domestication gene conferring the naked grain maize phenotype [66], displays strong linkage disequilibrium (Fig 4) consistently with previous observations [48]. Note that *TGA1* displayed no clear footprints of species-wide past selection despite the indisputable effect of a single amino acid substitution in the acquisition of the domesticated phenotype. Close interactions between *TGA1* and the nearby *NOT1* gene contribute to numerous pleiotropic effects and traits—including branching, kernel shape and size [67]. The complexity of effects and their interactions may produce a complex pattern of haplotypes.

### Conclusion

We scored over 22 million SNPs in American and European maize germplasm with high accuracy. This dataset helped refine a scenario consisting of two major introductions of maize to Europe. The range of latitudes in Europe represents a restricted subset of latitudes in the North America. We found that independent introduction sources and subsequent admixtures are keys to the spread of maize through Europe, mirroring the emergence of the admixed Corn Belt Dents in the US. This repeated pattern of admixture with newly introduced groups has contributed to adaptive innovations, paralleling recent findings in humans. Future work should help unravel the genome-wide specific contribution of parental groups to these admixed groups.

## Materials and methods

### Sampling and sequencing

We directed our sampling towards first-cycle inbred lines directly derived from landraces after a few generations of selfing. We established a list of all available first-cycle inbreds and gathered historical information to establish the name and geographic location of the corresponding landraces. Our sample included 48 of these lines. However, for the Mexican, Caribbean and South American lines, only a very small set of first cycle inbreds was available. We therefore used 9 Single Seed Descents (SSDs) and 10 Doubled Haploids (DHs) recently derived from Tropical landraces instead. Note that 8 of these SSDs were previously sequenced in [20]. The geographical locations of landraces from which the lines were derived is shown in Fig 1.

Our final sample encompassed 67 lines including 14 Corn Belt Dents (CBDs), 5 American Northern Flints (ANFs), 8 European Northern Flints (ENFs), 13 European Flints (EFs), 3 Spanish (Span), 5 Italians (It), 7 South Americans (2 and 5 respectively from the Northern and the Southern part), 7 Mexicans (5 lowlands and 2 highlands below/above 1500 meters) and 5 Caribbeans. We referred to Tropicals when combining South Americans, Mexicans and Caribbeans (19 Lines). Sample information with name, origin, provider, and status is available in S1 Table.

Genomic DNAs of all lines were extracted from fresh leaf tissue of a single plant using the Macherey-Nagel MaxiKit and sent to Integragen (Evry, France) for library construction and sequencing. Sheared total genomic DNA was used to generate Illumina paired-end libraries (500 bp insert size). Each library was paired-end sequenced (2 x 101 bp) with a target sequencing depth of 15x. Sequencing data volume for each line is detailed in S1 Table. DNA-sequencing reads from all lines were deposited in the European Nucleotide Archive (ENA) under the study accession number PRJEB14212.

### Mapping and SNP calling

All lines were aligned to the B73 v2. reference genome by combining Bowtie2 [68] and Stampy [69]. First Bowtie2 was used with default parameters, using a >98% identity threshold between each sample and the reference genome (parameter --score-min L,0,-0.12). In a second step, unmapped reads were used for mapping with Stampy (default parameters), which is a slower but more accurate aligner for insertion-deletion types of polymorphisms [69]. From SAM format outputs, we sorted and filtered out duplicates using Samtools V1.1 [70]. A total of 2,654 Gb of Illumina reads from the 67 samples were used to extract mpileup format with Samtools using properly mapped pairs only. We used mpileup raw counts to estimate genome coverage and read depth of each line. We considered as covered any base with at least 4 reads.

We performed genotype calling for each SNP with Varscan using the following parameters: Phred score above Q20, minimum coverage of 6 reads, α = 5%. For each sample, Varscan provides the number of reads that support variants at a given SNP. We utilized this information to define genotypes by performing a Likelihood Ratio Test (LRT) with one degree of freedom following [71] with slight modifications. Let n_1_ and n_2_ be the counts of most frequent and second most frequent nucleotide variants, we computed the likelihoods of a homozygote (genotype 1/1) or a heterozygote (1/2) as follows: L (1/1) ~ B(n_1_+n_2_,ε) and L (1/2) ~ B (n_1_+n_2_, 0.5), with the error rate ε = 0.01. If LRT was significant at α = 5%, we assigned the most likely genotype at the SNP.

### Customized filters and genotyping accuracy

After individual genotype calling, we considered the genotype information across samples to apply filters and improve genotype-calling accuracy. We retained only SNPs with two variants (biallelic). Our filters aimed at increasing proper SNP identification, and eliminating false heterozygosity created by misalignment of duplicated (paralogous) regions. We employed four criteria described below: multiple mapping, sequencing-depth variation, error rate, heterozygosity rate.

#### Multiple mapping

To avoid false genotype calling arising from genome redundancy, we retained genotypes (lines) for which the percentage of reads mapping to unique genomic positions was >90%. For reads aligned with Stampy, we used an alignment score>11 to define uniquely-mapped reads. We discarded positions that presented both ambiguous (with regards to multiple-mapped reads) and heterozygous genotypes.

#### Sequencing-depth variation

We eliminated genotyping information for all lines with a low sequencing-depth (<6) at a given position. In addition, we discarded positions with an average sequencing-depth > 28 across samples (28 represents the 99% average upper bound of the coverage). Such an elevated depth likely corresponds to missing portions in the reference genome and/or duplicated regions in our samples.

#### Error rate

For each genotype, we evaluated the error rate as the number of reads that differed from the called genotype, as given by the LRT (see above). We dropped positions for which (1) the probability of observing the number of error counts or more is above α = 5% given that B (n_1_+n_2_, ε), with ε = 0.01; (2) the distribution of the error counts among samples significantly differs from a Poisson distribution of parameter the average error count at the position (Kolmogorov-Smirnov test, α = 5%).

#### Heterozygosity rate

We eliminated heterozygous genotypes located in close vicinity (< 500 bp) to heterozygous insertion-deletions (indels). Local alignments are indeed known to be less accurate in regions encompassing such indels. When a heterozygote was detected among the 67 Lines, we retained only positions for which at least one homozygote of each variant was detected in other lines.

We extracted all positions that passed our stringent filters and determined the distribution of missing genotyping information across SNPs as well as the percentage of missing data per line. We annotated SNPs and classified them in two categories: within and outside genes. SNPs within genes were further subdivided into belonging to 5’UTR, 3’ UTR, exonic and intronic regions. Throughout the text, non-genic SNPs are defined as SNPs located >50kb away from annotated genes, and genic SNPs are defined as SNPs located within genes and 10kb on both sides of the genes.

### Genotype calling accuracy

To test the accuracy of our genotype calling we employed several approaches. First, we compared the rate of heterozygosity per line before and after applying our customized filters. We computed the per-line heterozygosity as the number of heterozygous sites divided by the number of heterozygous and homozygous sites for the alternative allele (allele differing from the reference). By considering alternative homozygotes only, this estimate compares each line to the reference independently from the level of diversity in the rest of the sample.

Second, we used 2 sets of high-confidence genes defined by [27] as “retained homoeologs” and “lost homoeologs”. The former encompasses genes with paralogs within the B73 genome, while the latter corresponds to single genes in B73 (with no paralogous copy). We expect no significant difference in the heterozygosity rate between those two sets of genes if our filters were efficient to discern true heterozygotes from homozygotes for different alleles at two duplicated loci. We retained genes with less than 30% of missing data—for a total of 2,788 genes with paralogs and 3,949 single genes—and computed the rate of heterozygosity per gene across our sample of 67 lines. We tested difference in the rate of heterozygosity between the two sets of genes using a bilateral Kolmogorov-Smirnov test (α = 5%).

Third, we extracted positions of the Illumina Maize SNP50k array from our HTS data to compare genotypes. For 42 lines, array genotyping was already available [72] but the DNAs used for genotyping came from different seed lots than the ones we used for HTS. Because first-cycle inbred lines exhibit residual heterozygosity, a substantial amount of heterozygous genotypes may differ between and within seed lots derived from the same line. We therefore also generated for another 20 lines new MaizeSNP50k genotyping from the exact same DNAs that served to produce the HTS data (S1 Table). We used Genome Studio (Illumina, v1) to call genotypes by applying the manually curated clustering of [73]. We first restricted our analysis to homozygotes both in Maize SNP50k array and HTS data. We calculated the error rate of genotype calling in our HTS data as the ratio of the number of discrepancies between the 2 datasets divided by the total number of genotypes evaluated, and considered either all 62 lines or the subset of 20 lines. Second, we used the subset of 20 lines to calculate errors rates for heterozygotes. We determined the false positive rate of HTS genotyping as the number of heterozygotes declared as homozygotes on the MaizeSNP50k divided by the number of heterozygotes in our HTS data. Conversely, we estimated the false negative rate as the number of heterozygotes found in the MaizeSNP50k but declared as homozygotes in our HTS data.

### Patterns of heterozygosity along chromosomes

We aimed at identifying regions with heterozygosity patterns deviating from the genome wide expectation across our sample of first-cycle inbreds and Single Seed Descent (SSD) lines (57). In order to do so, we employed a segmentation method on positions with MAF>5%. We considered the following statistical model. Let *X*_*ij*_ stand for the heterozygous status of line *i* at position *j*, with *X*_*ij*_ = 1 if the line is heterozygous, 0 otherwise. On a given chromosome, positions were assumed to be spread into *K* contiguous regions, each of these regions being characterized by a specific Heterozygosity Rate (*HR*), *i*.*e*. *X*_*ij*_~B(*p*_*k*_) if position *j* belongs to region *k*, where B(.) is the Bernoulli distribution and *p*_*k*_ is the *HR* in region *k*. The goal of the statistical analysis was to identify regions with associated *HR p*_*k*_ significantly higher than the genome wide HR. To this aim, a 2-step statistical analysis was performed.

In step 1 we applied a breakpoint detection procedure to jointly segment the heterozygous profiles and identify regions with homogeneous *HR*. When the number of regions *K* is known, the breakpoint detection problem boils down to finding the optimal splitting of the chromosome into *K* regions along with their associated *HR*{*p*_*1*_,…,*p*_*K*_}. A combination (splitting, *HR*{*p*_*1*_,…,*p*_*K*_}) is optimal if it achieves the best fit to the data, the fitting being measured through the likelihood. Identification of the combination (splitting, *HR* {*p*_*1*_,…,*p*_*K*_}) optimizing the likelihood was achieved through dynamic programming [74,75]. Since in practice the number of regions is unknown, we considered different values for *K*, ranging from 1 to 20,000. We further selected the optimal value K* using the model selection criterion proposed in [76].

In step 2, we applied a test procedure to identify regions with a *HR* significantly higher than the genome wide *HR*, noted *p*_*g*_. For a given region, the procedure consisted in testing H0 {*p*_*K*_ = *p*_*g*_} vs H1 {*p*_*K*_ > *p*_*g*_} using an exact Bernoulli test procedure. To control for multiple testing, a Benjamini Hochberg procedure [77] was performed at a nominal level of 5%. The true genome wide *HR* being unknown, we considered *p*_*obs*_ (0.41%) as the best estimate of *p*_*g*_, where *p*_*obs*_ is the genome wide averaged *HR* computed on the first-cycle inbred lines and SSDs (57). Note that *p*_*obs*_ was calculated as the number of heterozygotes divided by the number of heterozygotes and homozygotes. The previously described 2 steps procedure was applied to 57 lines (first-cycle inbreds and SSD lines).

For better visualization, we first merged independently significant (resp. non-significant) adjacent segments; second we removed segments containing a single SNP; and third we repeated the merging. In the end, we retained segments containing at least 5 SNPs. In the following we denoted segments displaying unexpected patterns of heterozygosity as heterozygous segments.

For heterozygous segments, we calculated the rate of heterozygosity in our subset of 10 HDs. We also determined the corresponding P-values using the test procedure described above.

We next asked whether heterozygous segments were enriched for deleterious mutations. To this purpose, we extracted genome wide genic SNPs and used SIFT4G_Annotator_v2.3 with the corresponding maize database [31] to identify both tolerated and deleterious variants. We tested for enrichment of deleterious variants within heterozygous segments compared with the rest of the genome using a χ^2^ test.

Because heterozygotes represented a small proportion of all genotypes, in all subsequent analyzes we considered heterozygotes as missing data and relied on a haploid model.

### Site frequency spectra and summary statistics

To evaluate the distributions of allele frequencies in our samples, we generated folded Site Frequency Spectra (SFS) using the DaDi (Diffusion approximation for Demographic inference) python library [78]. When constructing spectra, we projected down the sample size to account for missing data and differences in sample size. We generated folded SFS for (1) the entire sample of lines restricted to positions of the Maize SNP50k array, and (2) the entire sample (SFS projected down to 60 samples). Additionally, we retrieved all SNP positions available from *Tripsacum dactyloides* HTS data [20] to infer the ancestral versus derived status of our SNPs, and built an unfolded SFS considering the entire set of 67 Lines (projected down to 60 as above).

In order to further test the impact of introduction bottlenecks, we generated SFS on American and European lines (projected down to 29 samples) using non-genic SNPs. We compared our observed American SFS to the predicted “neutral” SFS obtained from a demographic model recently established by [28] for American landraces. This model incorporates a domestication bottleneck occurring 15,000 generations as well as a population expansion, and accounts for gene flow between wild and cultivated forms. We simulated sequences of 38 samples with MS [79] using the parameters indicated in Fig. 2 of [28] with an instantaneous expansion. We further established the corresponding SFS downsized to 29 samples. For non-genic SNPs, we also computed for American and European lines, genome-wide per-bp summary statistics with corrections for missing data [80]: *π*[81], Watterson’s *θ* [82], and Tajima’s *D* (*D*) [83]. In order to assess the significance of the difference in the amount of diversity between American and European samples, we estimated *π* on non-overlapping non-genic windows of 10kb along the genome (with less than 50% missing positions) and applied a pairwise Wilcoxon signed-rank test. Genetic diversity along chromosomes for the American and European samples as measured by *π* was computed on 50kb overlapping sliding windows with a step of 10kb. We used local linear polynomial fit for smoothing with lowess function of the R Package Stats with parameters 1/500 for smoother span.

### Genetic structuring

We assessed the population structure underlying our lines by combining different methods:

1. Genetic differentiation as measured by Fst. We estimated the Fst using non-genic SNPs from the equation of [84] for all pairwise comparisons between the 11 groups that were used to design our sample: Mexican highlands and lowlands, Caribbeans, Northern and Southern South Americans, American and European Northern Flints ANFs, ENFs), Corn Belt Dents (CBDs), European Flints (EFs), Spanish (Span), Italians (It). We chose the estimate of [84] because it is appropriate for small sample size, and values were calculated across all SNPs.
2. FastStructure v1.0 [85]. FastStructure minimizes deviations from Hardy—Weinberg between alleles within a predefined number of clusters (*K*). It provides admixture proportions of *K* clusters for each sample. We ran FastStructure using 500,000 non-genic SNPs uniformly distributed along the 10 chromosomes on the whole data set of 67 Lines, with *K* ranging from 1 to 10. A single run was carried out for each *K* using default parameters (convergence criterion: 10^−6^, choice of prior: simple and 10 cross-validations). To choose the appropriate number of clusters, we employed the model complexity that maximizes marginal likelihood. Additionally, we ran separate analyzes on American lines (CBDs, ANFs, Mexicans and Caribbeans) with *K* = 2, and European lines (All Europeans) with *K* = 2 to infer the proportion of admixture of two sets of lines with a recent history of hybridization, the Corn Belt Dents and the European Flints.
3. Principal components analysis (PCA). PCA allows samples projection on axes of variation reducing the data to a small number of dimensions. We used EIGENSTRAT v5 [86] on 500,000 non-genic SNPs uniformly distributed along the 10 chromosomes of the whole data set of 67 Lines, and retained the significant axes (α = 5%). We used the function DataEllipse of the R package ‘car’ v2.1–2 to draw 95% confidence ellipses of the Mahalanobis distance for genetic groups determined by FastStructure.
4. TreeMix analysis. Treemix models genetic drift to infer historical splits of populations deriving from an outgroup. But when populations are more closely related than modeled by the resulting bifurcating tree, TreeMix reconciliates the modelled covariance with the observed covariance by placing migration edges along the tree. These edges can originate either from existing populations or from unsampled, more basal populations. They are informative with regards to both the direction and the position of admixture relative to the divergence of the populations. We inferred admixture graphs using Treemix version 1.12 [87] using 109,580 non-genic SNPs (for which missing data did not exceed 20% of Lines in each population) oriented with the *Tripsacum dactyloides* outgroup. We considered all populations as defined in S1 Table. We tested 0 to 10 admixture events to build the graph. TreeMix was run on windows of 25 SNPs to account for linkage disequilibrium. We chose the graph corresponding to the first stabilized value of the likelihood, *i*.*e*. the value after which the likelihood does not substantially increase. In addition, we computed the three-population test (*f3* statistics) introduced by [88] to further validate admixture events inferred by TreeMix (threepop program of Treemix). *f3* is used to detect correlations in allele frequencies that are not compatible with evolution following a bifurcating tree. Negative value indicates that a population results from admixture of 2 other populations. A value of *Z*-score <−1.64 corresponds to a significant *P*-value at a 5% threshold.

### Detection of differential selection in genic regions

We employed 3 summary statistics to undertake genome scans for selection: Fst [84], XP-CLR [89] and Tajima’s *D*—thereafter *D* [83]. Fst and XP-CLR both measure genetic differentiation between two groups. XP-CLR also considers one of the two groups as a reference and incorporates local linkage disequilibrium information. *D* indicates deviation from the neutral SFS caused by demographic or selective processes.

We computed *D* and Fst values for each genic region encompassing > 7 SNPs and for which missing data did not exceed > 30% of lines in each group. For XP-CLR, genome-wide values were computed on sliding windows of 0.001 cM with 5000 bp steps. Individual SNPs were assigned a position along a genetic map by assuming uniform recombination between mapped markers. We down-weighted pairs of SNPs in high LD (*r*^2^> 0.7).

We considered the 5 groups defined by FastStructure as indicated in S1 Table. For Fst and XP-CLR we considered the following latitudinal comparisons: Northern Flints *vs* Corn Belt Dents, Corn Belt Dents *vs* Tropicals, Northern Flints *vs* Tropicals within the American continent; Northern Flints *vs* European Flints, European Flints *vs* SpanIt, Northern Flints *vs* SpanIt within the European continent. In addition, we performed longitudinal comparisons, American Northern Flints *vs* European Northern Flints, Corn Belt Dents *vs* European Flints, Tropicals *vs* SpanIt. We computed *D* within each of the 5 groups.

We attributed P-values for *D* and Fst as follows: (1) we determined *D* and Fst values for non-genic regions of similar range size as the genic regions; (2) we computed the P-value of each candidate gene as the proportion of non-genic regions with equal or higher Fst (resp. equal or lower *D*) than the value observed for this candidate gene. The same rationale was employed for XP-CLR. Non-genic windows were located at least 50kb away from genes.

We considered as putatively selected, genes passing the significance thresholds for all 3 statistics in at least one of the pairwise comparison that is, genes displaying: (1) a significant Fst (P-value<5%; and (2) a significant XP-CLR (P-value<5%) in at least one of the two reciprocal comparisons; and (3) a significant negative *D* (P-value<5%) within one of the 2 groups under comparison. These putatively selected genes are named Differentially-Selected (DS_) candidates thereafter.

### Detection of genes associated with latitude and longitude variation

We performed a Genowe Wide Association (GWA) analysis using all 67 lines and either latitude or longitude as phenotypes with FaST-LMM [90]. We considered for each SNP (MAF >5%) the following linear mixed model:

$$Y\;=\;X_{s}b_{s}\;+\;X_{m}b_{m}\;+\;ZU\;+\;E,$$

where *Y* is the vector of phenotypes (latitude or longitude), *X*_*s*_ is the structure matrix accounting for the membership in one of the 5 groups (NF, CBD, Tropical, EF, SpanIt, S1 Table), *b*_*s*_ is the vector of fixed structure effects, *X*_*m*_ is the genotype matrix at SNP *m*, *b*_*m*_ is the vector of allelic effects, *Z* is the identity matrix, *U* is a random effect accounting for the genetic background and *E* is an error term. Both *E* and *U* are assumed to follow a Gaussian distribution with mean 0 and covariance matrix *σ*^*2*^*I* and *σ*_*g*_^*2*^*K*, respectively, with *I* the identity matrix and *K*, the kinship matrix. The kinship matrix was estimated using the IBS estimator described in [91]. For each marker, the test H0: {*b*_*m*_ = 0} was performed. P-values were corrected to account for multiple testing using the BH procedure [77] to control the FDR at nominal level 5%.

### Gene ontology enrichment

Finally, we conducted a functional annotation of candidate genes using the MapMan Ontology (http://mapman.gabipd.org/web/guest/mapman). Considering only the subset of genes with a MapMan annotation, we tested for categories’ enrichment in candidate genes using a unilateral exact Fisher test. We performed this analysis on our set of DS_ candidates and GWA_candidates seperately.

### Selection on gene networks

To analyze selection at the network level, we employed the method of [30] with the following modifications. First, we retrieved a collection of 340 maize gene networks (pathways) available at http://maizecyc.maizegdb.org/ and selected a subset of 294 presenting more than 5 genes. For each annotated gene, we estimated pairwise Fst values and P-values as described above between all possible pairwise comparisons between the 5 groups (10 comparisons) and an additional comparison between American and European Northern Flints. P-values were further used to determine a normalized Z-score [30] following the qnorm function in R [92]. Because Z-scores are correlated with SNP number, genes were grouped into bins with similar number of SNPs. We performed a second normalization of the Z-score of each gene with the median of its corresponding bin. We calculated the network Z-score (SUMSTAT score as defined by [93]) as the sum of Z-scores across genes. SUMSTAT significance was evaluated by 1,000 resampling using all annotated genes with a number of genes equal to the network size.

## Supporting information

S1 Fig Distribution of maize reference genome coverage (B73) in our sample of 67 lines. (EPS)

S2 Fig Distribution of the percentage of missing data across lines at all SNPs. (EPS)

S3 Fig Folded site frequency spectra on the whole sample of 67 lines (HTS data) obtained using either MaizeSNP50 array positions only, or all positions. The former was computed on 33,046 positions (blue) and the latter on 10,813,686 positions (red). Only sites with less than 8 missing genotypes (≥60 genotyped lines) were conserved and the sample was projected down to 60 individuals.
(EPS)

S4 Fig Unfolded site frequency spectra on the whole sample of 67 lines obtained from High Throughput Sequencing data. The SFS is based on positions aligned to *T*. *dactyloides* which defines the allele ancestral state. The sample was projected down to 60 individuals.
(EPS)

S5 Fig FastStructure visualization of ancestry proportions at model complexity of *K* = 4 (A) and *K* = 5 (B). Each color represents a group. Vertical lines (individuals) are partitioned into colored segments whose length represents the admixture proportions from *K* groups. Classification of individuals according to the groups defined in the structuring analyzes (S1 Table) and further used for genome scan (Fig 2) are indicated below each graph: N = Northern Flints from America and Europe, E = European Flints, C = Corn Belt Dents, T = Tropicals, K = SpanIt.
(EPS)

S6 Fig Box plots of per-bp nucleotide diversity (*π*) in the 5 genetic groups used for selective scans. Nucleotide diversity is estimated on non-overlapping windows of 10kb along the genome (with less than 50% missing positions). N = Northern Flints from America and Europe, E = European Flints, C = Corn Belt Dents, T = Tropicals, K = SpanIt.
(EPS)

S7 Fig FastStructure visualization of ancestry proportions at model complexity of *K* = 2 for two admixed groups, the Corn Belt Dents (A) and the European Flints (B). Each color represents a group. Vertical lines (individuals) are partitioned into colored segments whose length represents the admixture proportions from 2 parental groups. Classification of individuals are indicated below each graph: N = American Northern Flints, C = Corn Belt Dents, T = Tropicals and K = SpanIt, E = European Flints, N = Northern Flints.
(EPS)

S8 Fig Variation of *π*/bp in the American (red) and European (blue) sample. Variation of *π*/bp was computed along chromosomes 2 to 10 from 50kb sliding windows.
(EPS)

S9 Fig Segregation of haplotypes in the vicinity of the *ZCN8* gene. Status of biallelic SNPs per line within each genetic group as inferred from Structure in a region encompassing the GWA_candidate, GRMZM2G095955, and the *ZCN8* gene.
(EPS)

S10 Fig Patterns of selection at the *ZCN5* gene. (A) Nei’s genetic diversity (H) averaged on overlapping sliding windows of 1,000 SNPs (1 SNP step) along chromosome 10 with positions indicated in kb (NFs in green, Tropicals in pink). (B) Status of biallelic SNPs per line within each genetic group as inferred from Structure is shown as well as pairwise LD computed from r^2^ from positions 113,529,804 to 114,530,887 bp. *ZCN5* position is indicated with a red box. (C) Zoom within the *ZCN5* genomic region with XP-CLR (dots) and *D* values (lines with the same colour code as in A) presented along with the genome-wide 5% quantile (green and pink dotted for *D* within NFs and Tropicals respectively, and black dotted line for XP-CLR between NFs and Tropicals).
(EPS)

S11 Fig Patterns of heterozygosity along chromosome 6 as determined by segmentation. (A) Rate of heterozygosity (%) among 57 lines. Positions along the chromosome are indicated in kb. Shaded area corresponds to centromere position. Horizontal bars designate the rate of heterozygosity per segment with grey and red corresponding to non-significant and significant segment respectively. (B) zoom within a specific region. Black crosses indicate rate of heterozygosity at each SNP. As in (A) non-significant and significant segments are indicated in grey and red respectively. Blue segments encompass less than 5 SNPs. Location of 4 genes within the region is shown by boxes underneath.
(EPS)

S12 Fig Distribution of P-values associated with heterozygosity rate in doubled haploids at 5,069 heterozygous segments defined from 57 lines. (EPS)

S13 Fig Distributions of the number of contributors to heterozygous segments as defined from 57 lines. A: segments significant in our sample of 57 lines (first-cycle inbreds and SSD lines)– 6,978 segments. B: subset of segments also significant in Doubled Haploids– 1,088 segments.
(EPS)

S1 Table List of plant material, alignment and sequencing statistics. (XLSX)

S2 Table Pairwise Fst matrix (P-value) computed between 11 groups defined a priori (cf. S1 Table). (XLSX)

S3 Table Description of Differentially Selected candidate genes (DS_candidates). (XLSX)

S4 Table Description of Genome Wide Association candidate genes (GWA_candidates). (XLSX)

S5 Table Enrichment test of MapMan ontologies for main and minor functional categories for DS_ and GWA_ candidates. (XLSX)

S6 Table Enrichment test for DS_candidates within gene networks. (XLSX)

S7 Table Description of segments displaying significantly elevated rate of heterozygosity (heterozygous segments). (XLS)
